# Bifidobacterium infantis-mediated HSV-TK/GCV therapy modulates the tumor microenvironment through site-specific phosphorylation of HIF-1α, mTOR, and PD-L1

**DOI:** 10.3389/fonc.2026.1849164

**Published:** 2026-06-22

**Authors:** Jiaming He, Yang Yuan, Jing Li, Yongping Ma, Dilong Chen

**Affiliations:** 1 Department of Biochemistry and Molecular Biology, Basic Medical College, Molecular Medicine & Cancer Research Center, Chongqing Medical University, Chongqing, China; 2 Laboratory of Stem Cells and Tissue Engineering, Department of Histology and Embryology, Chongqing Medical University, Chongqing, China; 3 Chongqing Key Laboratory of Development and Utilization of Genuine Medicinal Materials in Three Gorges Reservoir Area, Chongqing Three Gorges Medical College, Chongqing, China; 4 NMPA Key Laboratory for Quality Monitoring of Narcotic Drugs and Psychotropic Substances, Chongqing Institute for Food and Drug Control, Chongqing, China

**Keywords:** BF-TK/GCV, bifidobacterium infantis, bioinformatics, cancer, phosphoproteomics, transcriptomics

## Abstract

**Background:**

Bifidobacterium (BF)-mediated herpes simplex virus thymidine kinase (TK)/ganciclovir (GCV) therapy has shown antitumor activity, but its effects on protein phosphorylation remain unclear.

**Methods:**

Quantitative phosphoproteomic analysis was performed on HCT-116 tumor xenografts to identify phosphorylation changes induced by BF-TK/GCV treatment. Gene Ontology (GO) and Kyoto Encyclopedia of Genes and Genomes (KEGG) pathway enrichment analyses were conducted to explore the affected biological processes and signaling pathways. Immunohistochemistry (IHC) was used to validate the phosphorylation levels of key proteins. Additionally, RNA sequencing and IHC were applied to immunocompetent MC38 tumor-bearing mice to assess transcriptional changes and immune-related pathways.

**Results:**

Compared with the BF-TK group, BF-TK/GCV treatment resulted in 337 differentially abundant phosphopeptides (DAPs), and 108 DAPswere significantly altered relative to the BF/GCV group. GO analysis revealed enrichment in protein kinase activity, cell cycle regulation, and DNA replication. KEGG pathway analysis and IHC validation demonstrated that BF-TK/GCV differentially regulated the phosphorylation status of HIF-1α, mTOR, PKM2, and PD-L1 in a site-specific manner. Notably, IHC showed elevated immunoreactivity for several of these phosphoproteins in xenograft tumors, while phosphoproteomic comparisons also revealed decreased phosphopeptide abundance at other sites, indicating context-dependent modulation rather than uniform inhibition or activation. Notably, BF-TK/GCV suppressed tumor growth and significantly prolonged survival in tumor-bearing mice.

**Conclusions:**

Collectively, BF-TK/GCV does not uniformly inhibit or activate phosphorylation but rather reprograms the phosphoproteome in a site- and pathway-specific manner. These findings provide new insights into its therapeutic potential and suggest a promising strategy for cancer treatment.

## Introduction

According to GLOBOCAN 2022, there were approximately 20 million new cancer cases and 9.7 million cancer deaths worldwide (1), marking a continued rise from the 19 million cases and 10 million deaths reported in 2020 (2) and the 14.1 million cases and 8.2 million deaths reported in 2012 (3). By 2024, surveillance data from the United States alone projected approximately 2 million new cancer diagnoses and 600,000 deaths (4), underscoring the urgent need for novel, clinically actionable therapeutic strategies.

The landscape of cancer therapy has evolved from conventional approaches to multimodal regimens integrating molecular targeting, immune modulation, and bioengineering technologies. Innovations such as antibody-drug conjugates (ADCs), proteolysis-targeting chimeras (PROTACs), and oncolytic viruses have substantially expanded treatment options for molecularly stratified tumors, improving patient outcomes (5–7). Despite these advances, challenges persist, including drug resistance, systemic toxicity, and limited tumor specificity necessitating further exploration of alternative modalities.

Over the past two decades, gene therapy has emerged as a promising alternative for treating diverse human malignancies (8). Delivery vectors for such therapies are broadly categorized into viral, non-viral, and bacterial carriers (9, 10). Among these, Bifidobacterium (BF), a tumor-targeting, non-pathogenic, obligate anaerobic bacterium, has garnered attention for its reported ability to selectively colonize and proliferate within hypoxic tumor regions in previous studies (11), positioning it as a viable platform for oncotherapy (12). The herpes simplex virus thymidine kinase (HSV-TK)/ganciclovir (GCV) system represents one of the most extensively studied prodrug strategies and has been clinically explored for solid tumors, including melanoma. In this paradigm, BF-delivered TK is expressed within the tumor’s anaerobic core, where it metabolizes the prodrug GCV into monophosphorylated GCV. This intermediate is subsequently converted to GCV-triphosphate by endogenous cellular kinases. As a structural analog of deoxyguanosine triphosphate (dGTP), GCV-triphosphate either incorporates into elongating DNA strands or competitively inhibits DNA polymerase, ultimately triggering tumor cell death (13, 14).

Importantly, the HSV-TK/GCV system also exerts a “bystander effect”. Phosphorylated GCV metabolites can diffuse through gap junctions or be released from dying cells and taken up by neighboring tumor cells, thereby extending cytotoxicity to cells that do not express TK (15, 16). In addition to direct cell killing, the bystander effect may contribute to immune activation by releasing tumor antigens and damage-associated molecular patterns (DAMPs), potentially enhancing antitumor immune responses (17).

Transcriptome sequencing (RNA-seq) comprehensively characterizes the complete set of transcriptional outputs (including mRNAs, lncRNAs, circRNAs, etc.) within specific cells or tissues under defined physiological or pathological states. This high-throughput approach enables precise quantification of gene expression levels, discovery of novel transcripts and alternative splicing events, and elucidation of disease-associated gene regulatory networks, making it indispensable for identifying tumor biomarkers (18). Post-translational modifications (PTMs) orchestrate diverse cellular activities, among which phosphorylation is the most extensively studied. Under physiological conditions, phosphorylation dynamically regulates critical cellular processes, including growth, differentiation, apoptosis, and signal transduction. However, dysregulation of phosphorylation pathways is implicated in pathological consequences, particularly carcinogenesis. Aberrant phosphorylation-dephosphorylation cascades have been demonstrated in multiple oncogenic signaling pathways, such as receptor tyrosine kinases (RTKs), MAP kinases, cadherin-catenin complexes, and cyclin-dependent kinases (CDKs) (19).

Previous studies from our group have demonstrated that BF-TK/GCV can mediate apoptosis and suppress invasion/migration in bladder, colon, renal, and hepatocellular carcinoma cells through multiple mechanisms (12, 20, 21). However, the relationship between the antitumor effects of BF-TK/GCV and protein phosphorylation, as well as potential transcriptomic dysregulation in solid tumors, remains unclear. In this study, we employed TMT-based quantitative phosphoproteomics to investigate, for the first time, alterations in protein phosphorylation modifications in tumor cells following BF-TK/GCV treatment. Concurrent transcriptomic sequencing was performed to elucidate dysregulated cellular signaling pathways. Furthermore, we validated the expression of tumor biomarkers in two distinct animal models following BF-TK/GCV therapy.

## Materials and methods

### Cell and animal models

The human colorectal cancer cell line HCT-116 was obtained from the Cell Bank of the Chinese Academy of Sciences, while the murine colorectal cancer cell line MC38 was provided by the Molecular Medicine and Cancer Research Center of Chongqing Medical University, with both cell lines maintained in RPMI 1640 complete growth medium at 37 °C in a 5% CO_2_ atmosphere.

For *in vivo* studies, male BALB/c nude mice (4–6 weeks old, approximately 20 g, n=6) housed at the Laboratory Animal Center of Chongqing Medical University were subcutaneously injected with HCT-116 cells (1.0×10^8^ cells/mL) to establish xenograft models, followed by intratumoral administration of PBS, BF-TK (1.0×10^6^ CFU/tumor), BF/GCV (1.0×10^6^ CFU BF combined with 5.0 mg/kg GCV), or BF-TK/GCV (1.0×10^6^ CFU BF-TK with 5.0 mg/kg GCV) every 48 hours (22).

In parallel, immunocompetent C57BL/6J mice (4–6 weeks old, approximately 20 g, n=5 per group) from the same facility received subcutaneous injections of MC38 cells (2.0×10^5^ cells/mouse). The following treatment groups were included: PBS group: PBS only (i.v.) and PBS (i.p.) every 24 hours; pcDNA3.1-mCherry/GCV group: pcDNA3.1-mCherry (1.0×10^6^ CFU/mouse, i.v.) plus GCV (5.0 mg/kg, i.p.) every 24 hours; BF-TK/GCV group: BF-TK (1.0×10^6^ CFU/mouse, i.v.) plus GCV (5.0 mg/kg, i.p.) every 24 hours. All animal procedures were conducted in accordance with institutional guidelines under specific pathogen-free conditions.

### Protein extraction and quantification

HCT-116 tumor xenografts (3 of each group were randomly selected) treated with PBS, BF-TK, BF/GCV, or BF-TK/GCV were pulverized in liquid nitrogen using a pre-chilled mortar and pestle. Equal amounts (typically 100 mg) of cryo-ground tissue powder were homogenized in ice-cold lysis buffer (1% SDS, 8 M urea) supplemented with 1× phosphatase inhibitor cocktail (Roche). After 30 min of ice-cold lysis with intermittent vortexing, the homogenate was centrifuged at 15,000 × g for 30 min at 4 °C. The supernatant was collected and mixed with five volumes of pre-chilled acetone (-20 °C) by vigorous vortexing for protein precipitation, followed by overnight incubation at -20 °C. The precipitated proteins were pelleted by centrifugation (4 °C, 15,000 × g, 30 min), washed three times with 90% acetone to remove residual SDS, and subsequently resolubilized in fresh lysis buffer (8 M urea, 1% SDS with phosphatase inhibitors) through 2 min of probe sonication (40 kHz, 40 W, 5 sec on/off cycles). The solubilized protein solution was clarified by centrifugation (12,000 × g, 30 min, 4 °C), and the supernatant was subjected to protein quantification using a BCA assay kit (Pierce, Thermo Scientific) according to the manufacturer’s protocol, with absorbance measured at 562 nm on a microplate reader (BioTek). Protein concentrations were normalized across samples for downstream proteomic analysis.

### Protein alkylation and digestion

Protein samples were separated by SDS-PAGE for quality control prior to in-solution digestion. For proteomic processing, protein extracts were adjusted to 100 μL with lysis buffer (8 M urea, 1% SDS) and reduced with 10 mM tris(2-carboxyethyl)phosphine (TCEP) at 37 °C for 60 min. Alkylation was then performed by adding iodoacetamide (IAA) to a final concentration of 40 mM and incubating at room temperature in the dark for 40 min. Proteins were precipitated by adding ice-cold acetone (4:1 v/v) and storing at -20 °C for 4 h, followed by centrifugation at 10,000 × g for 20 min at 4 °C. The resulting protein pellets were resuspended in 100 μL of 100 mM triethylammonium bicarbonate (TEAB) buffer. Trypsin digestion was carried out at an enzyme-to-protein ratio of 1:25 (w/w) with incubation at 37 °C overnight (16–18 h). Digestion efficiency was verified by SDS-PAGE analysis before LC-MS/MS processing.

### Tandem mass tag labeling and fractionation

Phosphoproteomics was performed using TMT 10-plex reagents (Thermo Fisher, Cat. No. 90111). The experimental design included four groups (PBS, BF-TK, BF/GCV, and BF-TK/GCV) with three biological replicates per group, totaling 12 samples. To accommodate all 12 samples, two independent TMT 10-plex experiments were performed.

In the first TMT 10-plex experiment, the channels were assigned as follows: channels 126, 127N, and 127C for the three PBS replicates; channels 128N, 128C, and 129N for the three BF-TK replicates; channels 129C, 130N, and 130C for the three BF/GCV replicates; and channel 131 for a pooled reference sample. In the second TMT 10-plex experiment, channels 126, 127N, and 127C were used for the three BF-TK/GCV replicates, and the remaining channels were left empty except for channel 131, which again contained the same pooled reference sample to serve as a bridging channel for batch normalization. The pooled reference sample was prepared by mixing equal amounts of all 12 individual samples from both experiments.

The TMT reagent was removed from −20 °C storage and allowed to equilibrate to room temperature, followed by brief centrifugation. Anhydrous acetonitrile (ACN) was then added, and the mixture was vortexed and centrifuged. Each 100 μg aliquot of peptide was incubated with one tube of TMT reagent at room temperature for 2 hours. Subsequently, hydroxylamine was added to quench the reaction, and the mixture was incubated at room temperature for 15 minutes. Equal amounts of labeled peptides from each channel were then pooled within each TMT experiment and dried using a vacuum concentrator.

After LC-MS/MS acquisition, phosphopeptide intensities in each TMT channel were normalized to the reference channel (channel 131) within each experiment. Between-experiment batch effects were then corrected using “ComBat” as implemented in the “sva” R package. Normalized data from both experiments were merged for downstream differential analysis.

### Phosphopeptide enrichment

Phosphopeptides were enriched using a modified TiO_2_-based matrix-assisted selective enrichment technique. Lyophilized peptide samples were first reconstituted in 50 μL of 80% acetonitrile (ACN) containing 0.1% trifluoroacetic acid (TFA). The enrichment column was pre-equilibrated with Buffer A (80% ACN, 0.4% TFA) and then conditioned with Buffer B, consisting of 250 mg/mL lactic acid, 60% ACN, and 0.3% TFA. The reconstituted peptides were mixed with Buffer B and loaded onto the column, with the loading step repeated twice to ensure optimal binding. After multiple washes with Buffer B and Buffer A, phosphopeptides were sequentially eluted using 5% ammonium hydroxide and 5% pyrrolidine solutions. The combined eluates were acidified with 20% TFA, desalted using StageTips, and dried in a vacuum concentrator for subsequent mass spectrometry analysis.

### Liquid chromatography–tandem mass spectrometry analysis

**Figure 1:**
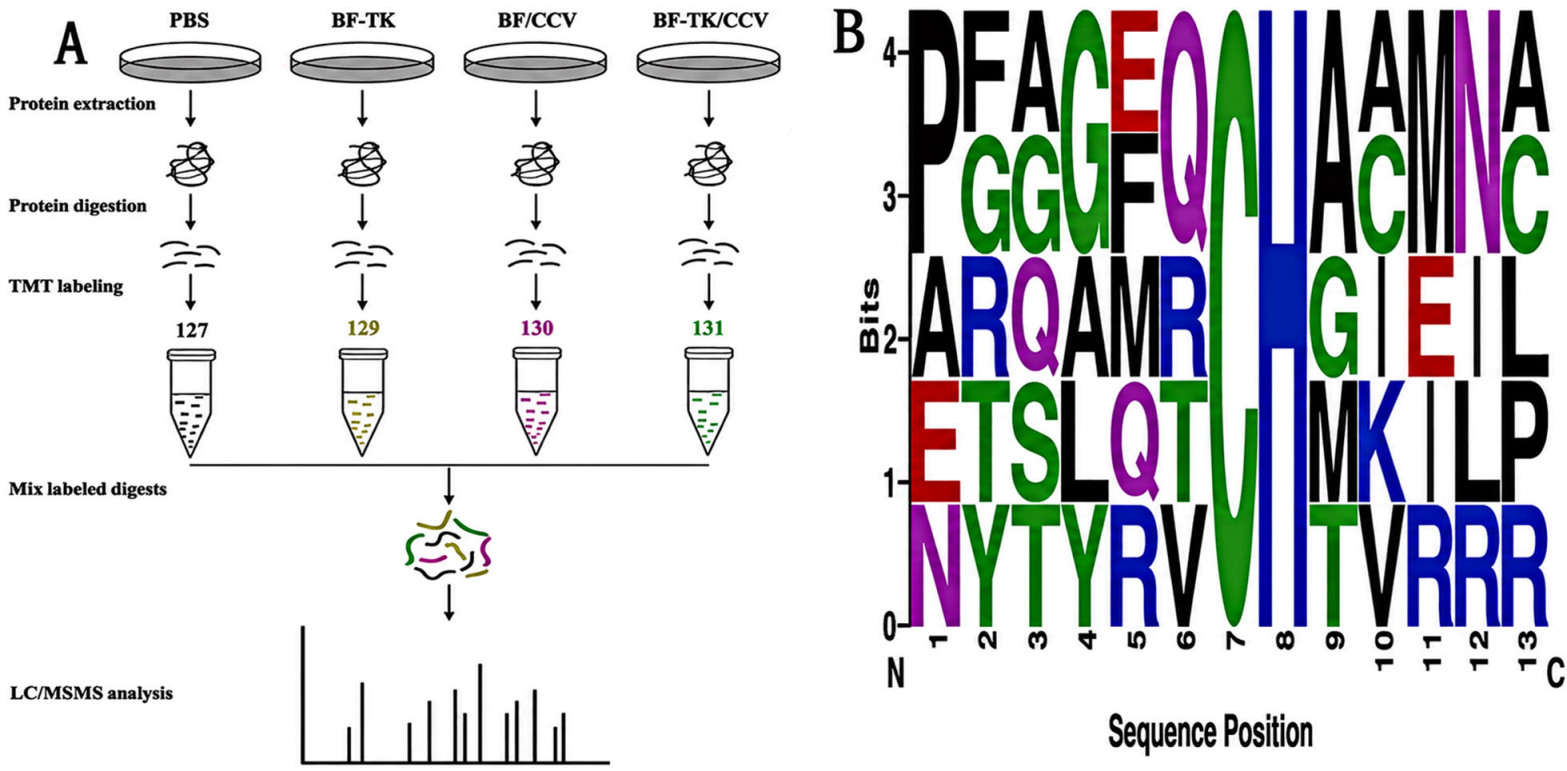
Overview of phosphoproteomics analysis. **(A)** Experimental workflow. HCT-116 xenograft tumor tissues from treated mice were analyzed by TMT 10-plex quantitative phosphoproteomics. Labeled peptides were enriched using titanium dioxide. **(B)** Motif analysis of phosphorylation sites. The x-axis shows peptide position relative to the modified residue; the y-axis shows information content (bits). Amino acids are arranged in descending order of information content at each position.

Protein digestion was performed according to standard protocols. The resulting peptide mixtures were labeled with a 10-fold molar excess of TMT reagent (Thermo Fisher, Cat. No. 90111) according to the manufacturer’s instructions. To increase proteome coverage, the labeled peptides were fractionated using ultra-performance liquid chromatography (UPLC) on a BEH C18 column (1.7 µm, 2.1 mm × 150 mm, Waters, USA). Subsequently, the labeled peptides were analyzed by tandem mass spectrometry using an online nanoflow liquid chromatography system (Easy-nLC 1200, Thermo Fisher Scientific, USA) coupled to an Orbitrap mass spectrometer (**Figure 1A**).

### Raw data interpretation

The MS/MS raw data were analyzed using MaxQuant software (version 2.3.2) (23). To accurately identify peptides from HCT-116 tumor xenografts, the data were searched against a combined database of the UniProt Human proteome (Taxon ID: 9606) to account for both tumor cells and the host stromal components. The search parameters were set as follows: trypsin was specified as the digestion enzyme, allowing up to two missed cleavages; the precursor mass tolerance was 20 ppm, and the fragment mass tolerance was 0.02 Da. Carbamidomethylation of cysteine residues and TMT labeling of peptide N-termini and lysine side chains were set as fixed modifications, while methionine oxidation and phosphorylation (S/T/Y) were included as variable modifications. Both peptide and protein identifications were filtered using a false discovery rate (FDR) threshold of < 0.01. Phosphopeptides with an absolute fold change (FC) > 1.20 or < 0.83 and a FDR < 0.05 were considered DAPs. We use the term “DAPs” throughout because phosphopeptide abundance changes were not normalized to total protein levels; therefore, increased or decreased abundance may reflect changes in protein abundance, phosphorylation occupancy, or both. For protein-level quantification, at least one unique phosphopeptide was required.

### Selection and confirmation of phosphosites for IHC validation

Four phosphosites were selected for IHC validation: HIF-1α S641/643, PKM2 Y105, mTOR S2448, and PD-L1 S283. These sites were selected based on the following criteria: (i) each was significantly differentially abundant in the BF-TK/GCV vs. control comparisons (|FC| > 1.20, FDR < 0.05); (ii) each had a high localization probability (>0.99) in the MaxQuant output; (iii) they are functionally relevant to cancer-related pathways (HIF-1 signaling, glycolysis, PI3K/AKT/mTOR, and immune checkpoint regulation). All four sites were confidently identified in our TMT-based phosphoproteomic dataset from HCT-116 xenograft tumors.

### Motif analysis

Motif analysis was performed using the MoMo software (version 5.4.1, http://meme-suite.org/tools/momo), which employs the motif-x algorithm for sequence model analysis (24). In this analysis, amino acids at specific positions are represented by modification-13-mers. Phosphorylation sites were identified with 6 residues upstream and 6 residues downstream of the modification. The background database parameter was set to all protein sequences. The minimum occurrence threshold was set to 5. Statistical significance was determined with a p-value threshold of < 0.000001. The “Simulate original motif-x” option was selected, with all other parameters set to their default values (19).

### Functional and network analysis

Overlapping DAPs among groups were explored and visualized using the data interaction and visualization platform, Evenn (http://www.ehbio.com/test/venn/) (25). Gene Ontology (GO) analysis was performed using R (version 4.4.1) to assess enrichment in biological process (BP), molecular function (MF), and cellular component (CC). KEGG pathway analysis was conducted to identify enriched pathways, with a significance threshold of P < 0.05. Protein-protein interactions (PPI) were assessed using the Search Tool for the Retrieval of Interacting Genes (STRING, version 11.5, https://string-db.org/) (26) and visualized in Cytoscape (version 3.9.1, http://www.cytoscape.org/) (27) with a medium confidence score (score = 0.4). Additionally, the cytohubba plugin (Cytoscape) was used to calculate the degree of connectivity and identify the top 10 hub proteins (28). The highest-scoring clusters were analyzed using the MCODE plugin (29), and pathway enrichment results were visualized using Venn diagrams via the “Evenn” package.

### Phosphorylation site data analysis

The analysis of the raw data was initially limited to pairwise comparisons among four groups, which restricted the depth of information interpretation. To address this, the raw data were reanalyzed using MaxQuant software, followed by subsequent processing with its companion tool Perseus (version 2.1.1.0) (30). In Perseus, the data obtained from MaxQuant analysis were imported, and the adjusted protein abundances between groups were selected as categorical variables. Phosphorylation sites and proteins were designated as the primary variables, with protein IDs and phosphorylation site IDs serving as information variables. A series of data preprocessing steps was then performed, including the removal of contaminant proteins and reverse database sequences, Log2 transformation of the data, and replacement of missing values with normal distribution imputation. The experimental design included three experimental groups (BF-TK, BF/GCV, BF-TK/GCV) and one control group (PBS), with three biological replicates per group. Appropriate annotations were added to the sample columns. To identify differentially phosphorylated sites, two-sample t-tests were conducted, yielding results from six pairwise comparisons. Based on the “difference” column in the exported data, further comparison of the differential phosphorylation sites between groups was performed.

### Transcriptome sequencing analysis

Half of the freshly obtained tumor tissue (3 of each group were randomly selected) was quickly dissected and placed on dry ice, labeled, and then sent to Shanghai Wei Huan Biological Technology Co., Ltd. for transcriptome sequencing data acquisition.

### Differential analysis and KEGG, GO analysis

Raw RNA-seq reads were quality-trimmed using Trimmomatic (version 0.39) and aligned to the mouse reference genome (GRCm39) using STAR (version 2.7.10a). Gene-level raw counts were obtained using featureCounts (version 2.0.3). Differential expression analysis was performed using DESeq2 (version 1.38.3) in R (version 4.4.1). The Wald test was used for hypothesis testing, and multiple testing correction was applied using the Benjamini-Hochberg false discovery rate (FDR) method. Differentially expressed genes (DEGs) were defined as |log2 fold change| ≥ 1 and FDR < 0.05. The differentially expressed genes (DEGs) were then imported, and KEGG and GO analyses were conducted using the “clusterProfiler”, “ggplot2”, “DOSE”, and “stringi” packages (31–33).

### Antibodies and immunohistochemistry

The following is the antibody catalog: Phospho-PKM2-Y105 Rabbit pAb (Abclonal, Wuhan, China, AP0924), phospho-mTOR-Ser2448 (Abmart, Shanghai, China, T56571), phospho-HIF1A-Ser641/Ser643 (Affinity, Jiangsu, China, AF0062), CD80 Rabbit pAb (Abclonal, Wuhan, China, A16039), CD86 antibody (Abmart, Shanghai, China, T55238), Phospho-PD-L1/CD274-S283 polyclonal antibody (Biorbyt, Cambridge, United Kingdom, orb1722637). All antibodies were administered according to the manufacturer’s instructions.

Immunohistochemistry (IHC) was performed on 4-μm formalin-fixed paraffin-embedded (FFPE) tum Paraffin-embedded tissue sections (4 μm) were deparaffinized with xylene and hydrated with gradient ethanol, and then heat-repaired with sodium citrate buffer (pH 6.0) at 95 °C for 20 minutes. Endogenous peroxidase was blocked for 15 min, and 5% BSA for 30 min at room temperature. Subsequent sections were incubated with primary antibodies overnight at 4 °C. After washing with PBS, HRP-labeled secondary antibodies were incubated at room temperature for 1 h, stained with DAB, and nuclei were counterstained with hematoxylin. Images were acquired using a light microscope (Shanghai, China, Nikon Eclipse E100) and quantified by ImageJ software (34). Each repeat was performed as a separate, independent experiment.

### Statistical analysis

Statistical analysis was performed using GraphPad Prism software (San Diego, CA., version 9.0.2). One-way analysis of variance (ANOVA) was used for comparisons among multiple independent variables. *Post-hoc* pairwise comparisons were conducted using the LSD method. Differences were considered significant at a p-value of < 0.05.

## Results

### Phosphoproteomics data analysis of gene therapy

To better understand the mechanism by which BF-TK/GCV inhibits tumors through protein phosphorylation, HCT-116 xenograft were treated with PBS, BF-TK, BF/GCV, and BF-TK/GCV, and the cell lysates were subjected to mass spectrometry analysis using the 10-plex TMT quantitative phosphoproteomics technology to detect changes in protein phosphorylation (**Figure 1A**; **Supplementary Figure 1**). The results of the motif analysis were presented as a probability plot, showing the amino acid composition surrounding the target modification sites (**Figure 1B**). A 13-amino acid sequence probability distribution was displayed. The first position at the N-terminus exhibited a highly conserved P residue, followed by positions 2–6, which showed diversity (R, G, F, Q, T, etc.), and positions 7–8 displayed highly conserved G and H residues with higher information content. The C-terminus (positions 9–13) exhibited higher amino acid diversity (A, C, M, N, etc.). The letters in the figure represent the relative frequency of amino acids at each position, with information content measured in Bits.

**Figure 2:**
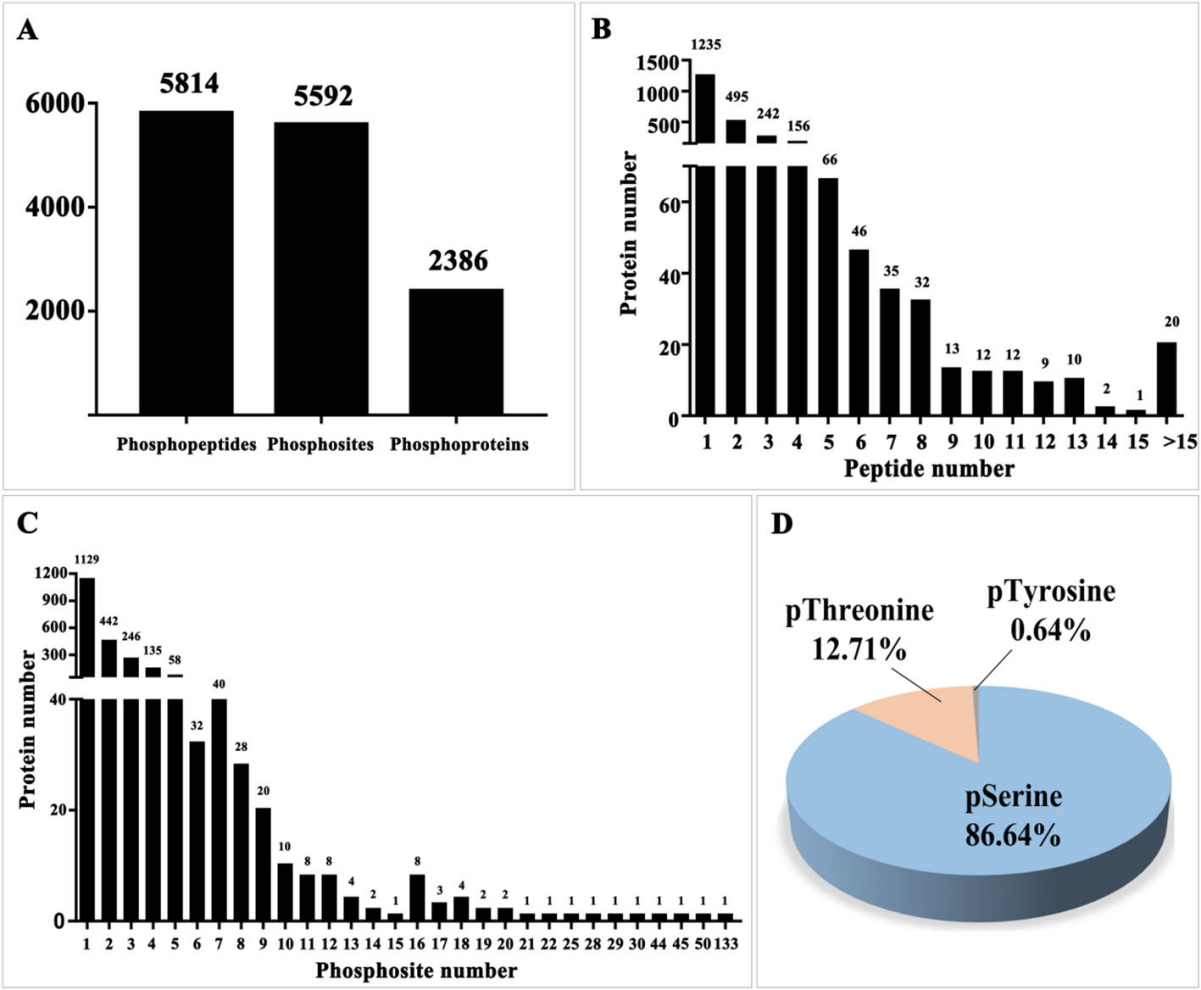
Phosphoproteomics analysis of peptide and residue distribution. **(A)** Number of quantified phosphopeptides, phosphorylation sites, and phosphoproteins. **(B)** Distribution of phosphopeptide counts per phosphoprotein. **(C)** Distribution of phosphorylation site counts per phosphoprotein. **(D)** Distribution of phosphorylation residues: serine (pS), threonine (pT), and tyrosine (pY).

Through proteomics analysis, 5814 unique phosphopeptides and 5592 unique proteins with increased phosphopeptide abundance (PIPAs) were identified (**Figure 2A**), of which 2386 PIPAs (51.76%) contained only a single phosphopeptide, 495 (20.75%) contained two phosphopeptides, and 656 (27.49%) contained three or more phosphopeptides (**Figure 2B**). Among the 2386 quantified PIPAs, 1129 (47.32%) contained a single phosphorylation site, 442 (18.52%) had two phosphorylation sites, and 815 (34.16%) had more than two phosphorylation sites (**Figure 2C**). The distribution of phosphoserine (pS), phosphothreonine (pT), and phosphotyrosine (pY) sites was 86.64% (4845), 12.71% (711), and 0.64% (36), respectively (**Figure 2D**). Notably, serine phosphorylation (pS) exhibited the highest frequency.

### Identification of DAPs

**Figure 3:**
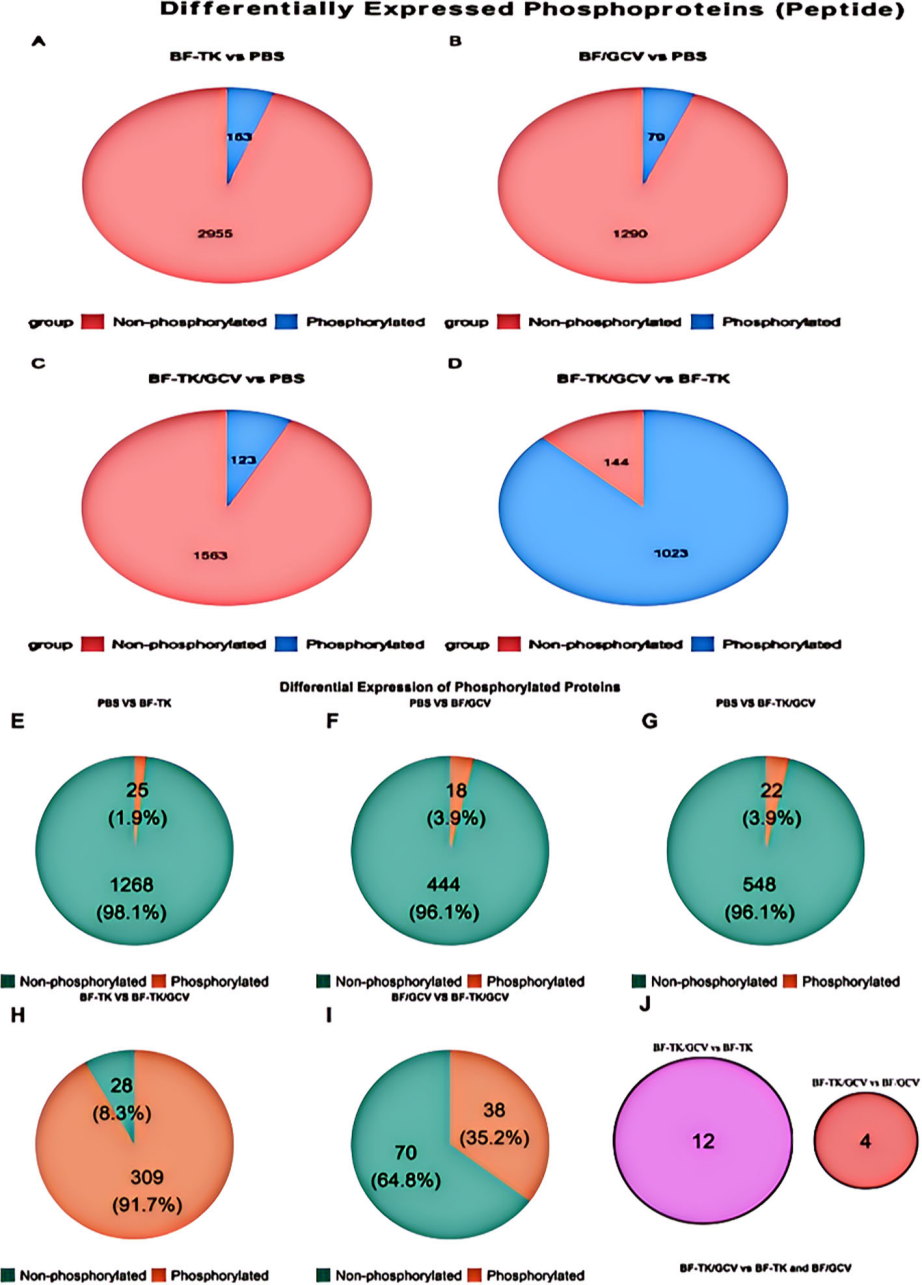
Expression analysis of identified differential phosphopeptides. **(A–D)** Pie charts show the number of phosphopeptides with increased abundance (red) and decreased abundance (blue) in the BF-TK, BF/GCV, and BF-TK/GCV groups compared to PBS, as well as in the BF-TK/GCV group compared to the BF-TK group. **(E–I)** Pie charts display the number of proteins with increased phosphopeptide abundance (orange) and decreased phosphopeptide abundance (green) in the BF-TK, BF/GCV, and BF-TK/GCV groups compared to PBS, as well as in the BF-TK/GCV group compared to the BF-TK group. **(J)** Pie chart shows that 12 and 4 PIPAs exhibit similar regulatory roles in the BF-TK/GCV vs BF-TK and BF-TK/GCV vs BF/GCV comparisons, respectively.

The criteria for selecting significantly DAPs were set as FC > 1.20 or < 0.83. After screening, visualization was performed using the ggplot2 package. We first observed the differentially expressed peptides after enrichment. Compared to the PBS group, the BF-TK group had 3118 DAPs(163 PIPAs, 2955 PDPAs) (**Figure 3A**), the BF/GCV group had 1369 DAPs (79 PIPAs, 1290 PDPAs) (**Figure 3B**), and the BF-TK/GCV group had 1686 DAPs (123 PIPAs, 1563 PDPAs) (**Figure 3C**). Compared to the BF-TK group, the BF-TK/GCV group showed 1167 DAPs (1023 PIPAs, 144 PDPAs) (**Figure 3D**).

Next, we further explored the differential expression ofPIPAs. Compared to the PBS group, the BF-TK group showed 1293 DAPs (25 PIPAs, 1268 PDPAs) (**Figure 3E**), the BF/GCV group showed 462 DAPs (18 PIPAs, 444 PDPAs) (**Figure 3F**), and the BF-TK/GCV group showed 570 DAPs (22 PIPAs, 548 PDPAs) (**Figure 3G**). Compared to the BF-TK group, the BF-TK/GCV group showed 337 DAPs (309 PIPAs, 28 PDPAs) (**Figure 3H**). Compared to the BF/GCV group, the BF-TK/GCV group showed 108 DAPs (38 PIPAs, 70 PDPAs) (**Figure 3I**). Furthermore, in the BF-TK/GCV vs BF-TK and BF-TK/GCV vs BF/GCV comparisons, 12 and 4 PIPAs exhibited similar regulatory roles, which may indicate that they are important proteins in the action of BF-TK/GCV (**Figure 3J**).

### Construction of pathway and PPI networks

**Figure 4:**
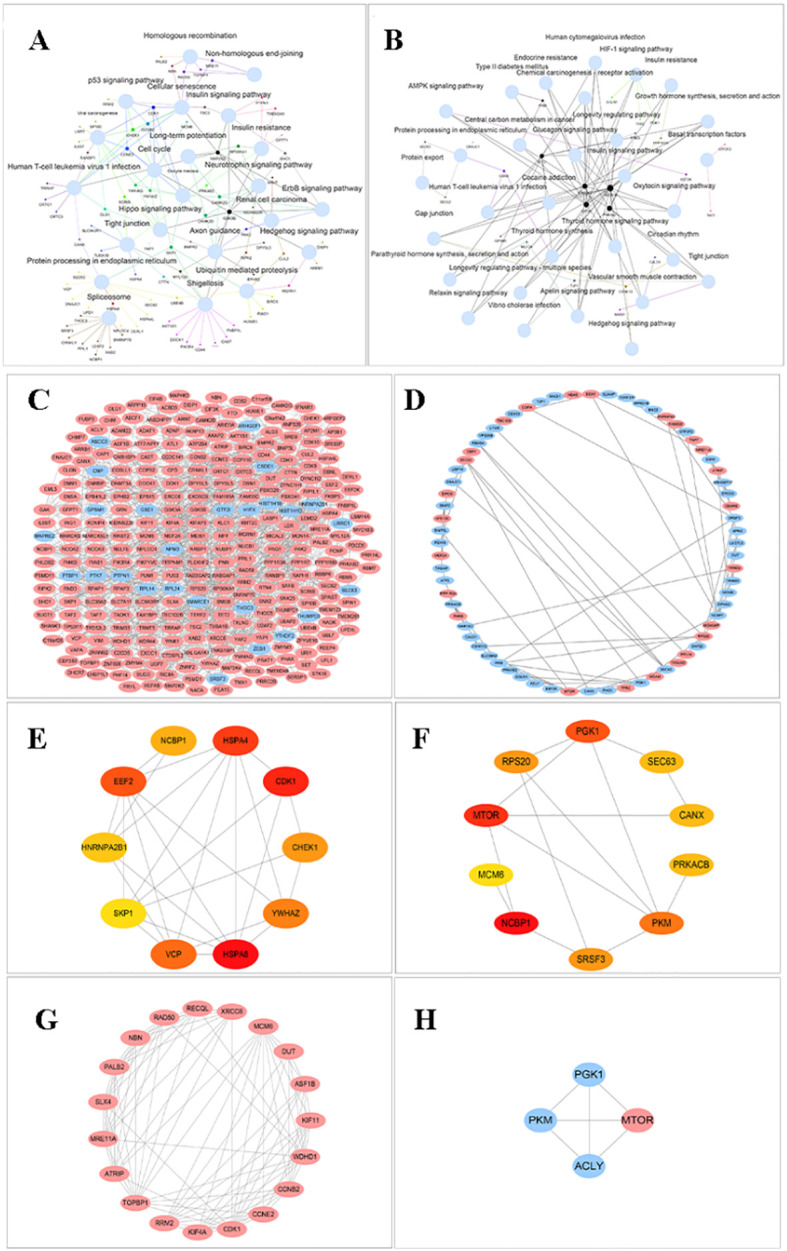
Protein-protein interaction network and subnetwork of DAPs. **(A, B)** Pathway-protein association networks for BF-TK/GCV vs. BF-TK **(A)** and BF-TK/GCV vs. BF/GCV **(B)**. Small nodes: proteins; large blue nodes: KEGG pathways. **(C, D)** PPI networks of DAP-associated proteins (STRING database). Red: increased phosphopeptide abundance; blue: decreased abundance. **(E, F)** Hub protein subnetworks identified by CytoHubba (darker red indicates higher rank). **(G, H)** Highest-scoring clusters identified by MCODE.

Venn network analysis was conducted to explore the relationship between DAPs and significantly enriched KEGG pathways (P < 0.05). As shown in **Figure 4A**, among the 80 proteins in 22 pathways, MAP2K2 and GSK3B had the highest connectivity in the BF-TK group. **Figure 4B** illustrates the relationships between 30 pathways and 25 proteins in the BF-TK/GCV versus BF/GCV comparison group. Several genes, including MAP2K2, MTOR, ATF2, PRKACB, and PRKAB2, were involved in multiple pathways. These highly connected proteins are predominantly involved in the HIF-1 and AMPK signaling pathways.

Additionally, we focused on the PPI network and hub genes in both groups. The PPI network was constructed based on the STRING database to elucidate protein-protein interactions (**Figures 4C, D**). In these PPI networks, the degree centrality method in the CytoHubba plugin was used to identify the top 10 PIPAs as hub proteins. In the BF-TK/GCV versus BF-TK comparison group, the top 10 hub proteins were HSPA8, EEF2, HSPA4, VCP, SKP1, HNRNPA2B1, YWHAZ, CDK1, CHEK1, and NCBP1 (**Figure 4E**). In the BF-TK/GCV versus BF/GCV comparison group, the top 10 hub proteins were PKM, MTOR, PGK1, NCBP1, RPS20, SRSF3, SEC63, CANX, MCM6, and PRKACB (**Figure 4F**).

Furthermore, clustering within the network was performed using MCODE to identify highly interconnected regions, with the highest-scoring clusters shown in **Figures 4G, H**. In the BF-TK/GCV versus BF-TK group, the cluster consisted of upregulated PIPAs such as NBN, RAD50, RECQL, XRCC6, MCM6, DUT, ASF1B, KIF11, WDHD1, CCNB2, CCNE2, CDK1, KIF4A, RRM2, TOPBP1, ATRIP, MRE11A, SLX4, and PALB2. The cluster in the BF-TK/GCV versus BF/GCV group included PKG1, PKM, ACLY, and MTOR. Some of the hub proteins and PIPAs in the clusters, such as PKG1 and MTOR, were involved in the HIF-1 and AMPK signaling pathways.

### Functional enrichment analysis

**Figure 5:**
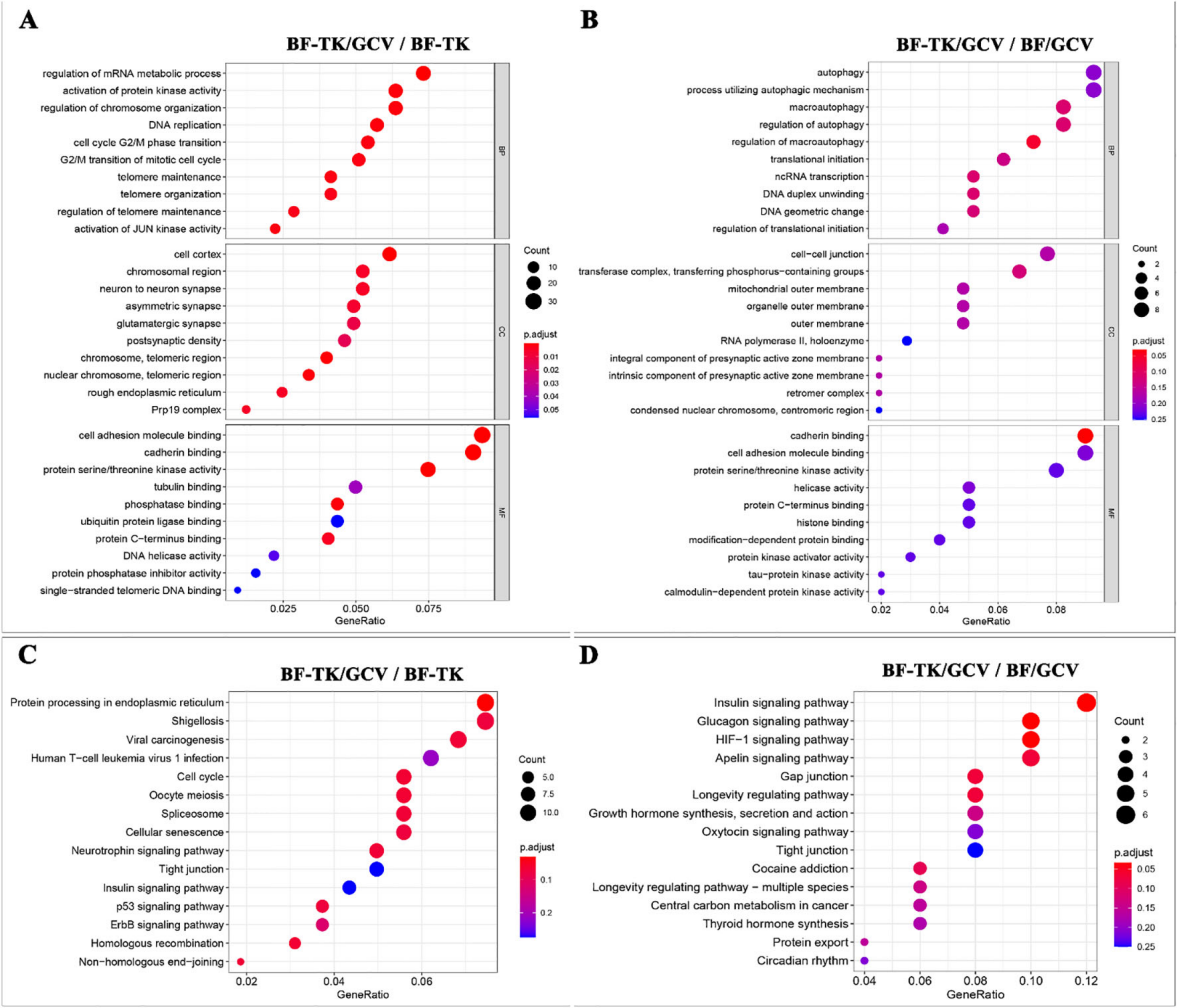
GO and KEGG enrichment analysis. **(A)** Gene Ontology (GO) analysis of DAPs in the BF-TK/GCV vs. BF-TK, and **(B)** BF-TK/GCV vs. BF/GCV comparison groups, showing the top ten enriched biological processes (BP), cellular components (CC), and molecular functions (MF). **(C)** The top 15 enriched KEGG pathways are displayed in BF-TK/GCV vs. BF-TK, and **(D)** BF-TK/GCV vs. BF/GCV comparison groups.

To gain a deeper understanding of the biological classification between the three treatment groups, we used the BF-TK and BF/GCV groups as controls and analyzed the differential DAPs of the BF-TK group. In the BF-TK group, a total of 1943, 367, and 299 significant enrichments were identified in Biological Process (BP), Cellular Component (CC), and Molecular Function (MF), respectively. The top 10 enriched GO terms are shown in **Figure 5A**. Simultaneously, we performed GO enrichment analysis on the 108 DAPs from the BF/GCV group, which revealed 118, 32, and 22 significant enrichments in BP, CC, and MF, respectively (**Figure 5B**).

We also performed KEGG pathway enrichment analysis. In the BF-TK group, 48 pathways were significantly enriched, with DAPs primarily involved in the cell cycle and cellular senescence (**Figure 5C**). In the BF/GCV group, 28 pathways were significantly enriched, and KEGG pathway analysis revealed that DAPs are mainly involved in the HIF-1 and AMPK signaling pathways (**Figure 5D**).

### Visualization of differential phosphorylation sites of proteins

The exported data were further analyzed using R (version 4.4.1). Based on the results from Proteome Discoverer (Thermo Scientific, version 2.1), proteins with missing phosphorylation sites were filtered out. By comparing the common features of protein phosphorylation across groups, 34 proteins with decreased phosphopeptide abundance (PDPAs) and 257 PIPAs were identified.

**Figure 6:**
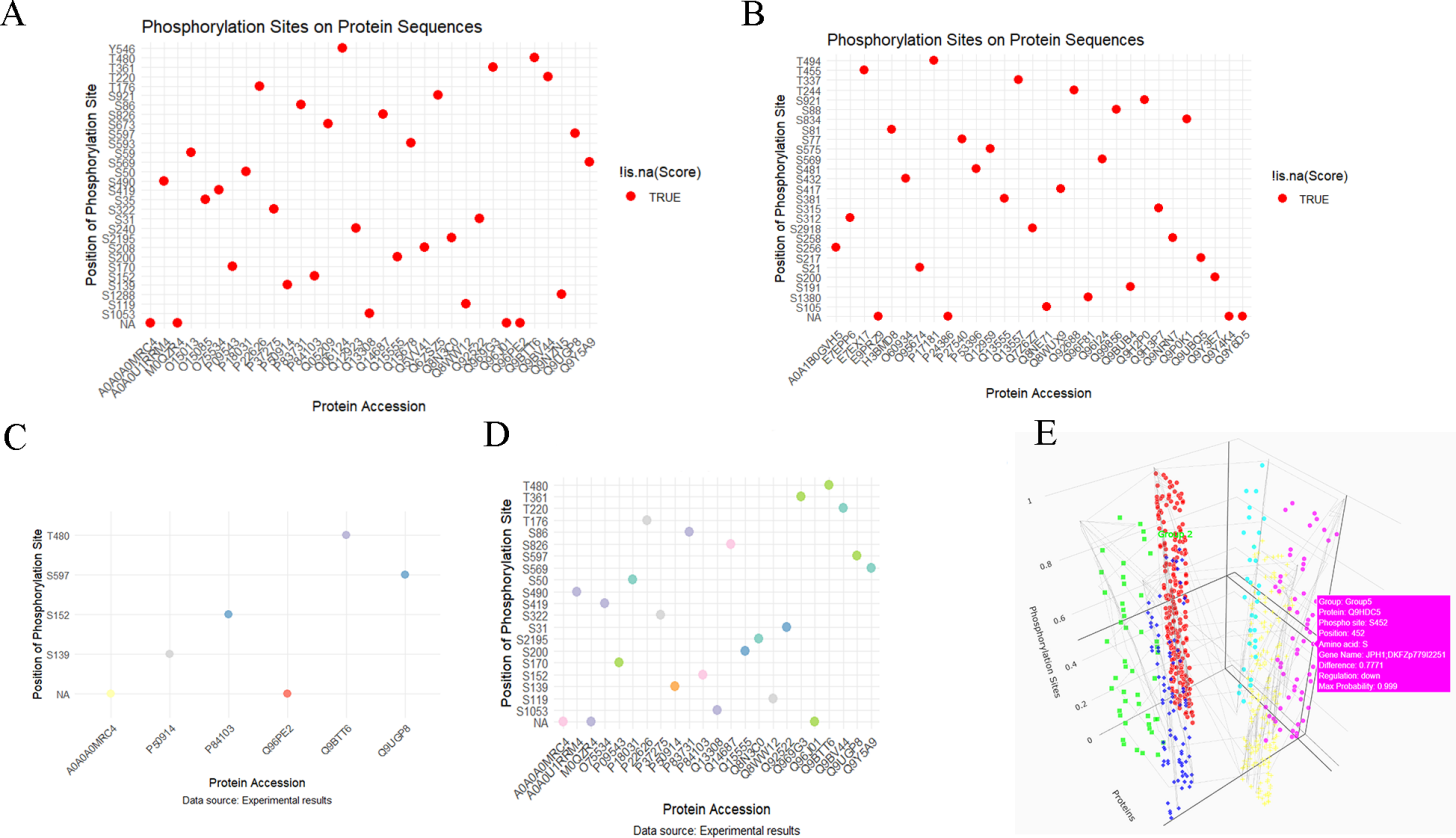
Spatial distribution of PIPAs. **(A)** Distribution of phosphorylation sites along protein sequences (x-axis: proteins; y-axis: site coordinates). **(B)** Top 30 significantly differential phosphorylation sites. **(C, D)** Proteins with decreased phosphopeptide abundance in BF-TK/GCV vs. BF-TK **(C)** and BF-TK/GCV vs. BF/GCV **(D, E)** Three-dimensional visualization of phosphorylation sites across six comparison groups. Color intensity indicates differential abundance; gray lines connect shared sites between groups.

The top 30 dephosphorylated and PIPAs are shown in **Figure 6A**. The prediction probability scores for these phosphorylation sites all exceed 0.99, indicating high confidence in these sites. Additionally, the top 30 most significantly different phosphorylated sites between groups were selected for visualization (**Figure 6B**).

In the BF-TK/GCV vs. BF-TK comparison group, the PDPAsinclude MRC4, RPL14, SRSF3, ARHGEF17, LRRC8B, and SEC63 (**Figure 6C**). In the BF-TK/GCV vs. BF/GCV comparison group, the PDPAs include MRC4, KIAA0391, RRM2, CKAP4, ARHGEF1, ASCC3, CSDE1, PCBP4, CNP, H1FX, PTPN1, SMCR8, HNRNPA2B1, THOC6, ZNF516, SEC63, SRSF3, YTHDF2, and LSM4 (**Figure 6D**).

Traditional 2D plots have limitations in presenting information, so we used the plotly package to create a more informative 3D visualization (**Figure 6E**). Data points with a differential value greater than 0.5 were selected to plot the three-dimensional spatial distribution of phosphorylation sites. The figure includes six groups of data representing phosphorylation data of DAPs from six comparison groups. Phosphorylation protein data were further reduced, and this figure is used to statistically display the information of protein phosphorylation sites, providing an intuitive display of common phosphorylation sites across the groups. The visualization highlights the relationships between these sites through connecting lines. For example, in the BF/GCV vs. BF-TK/GCV comparison group, the phosphorylation site of NCOR2 protein is S1858, with amino acid modification at S. The regulatory gene of NCOR2 protein is ncor2, with a difference value of 2.027215958, indicating a downregulation of the phosphorylation protein, and the reliability of the phosphorylation site is 0.999.

### Phosphorylation site association effect analysis

**Figure 7:**
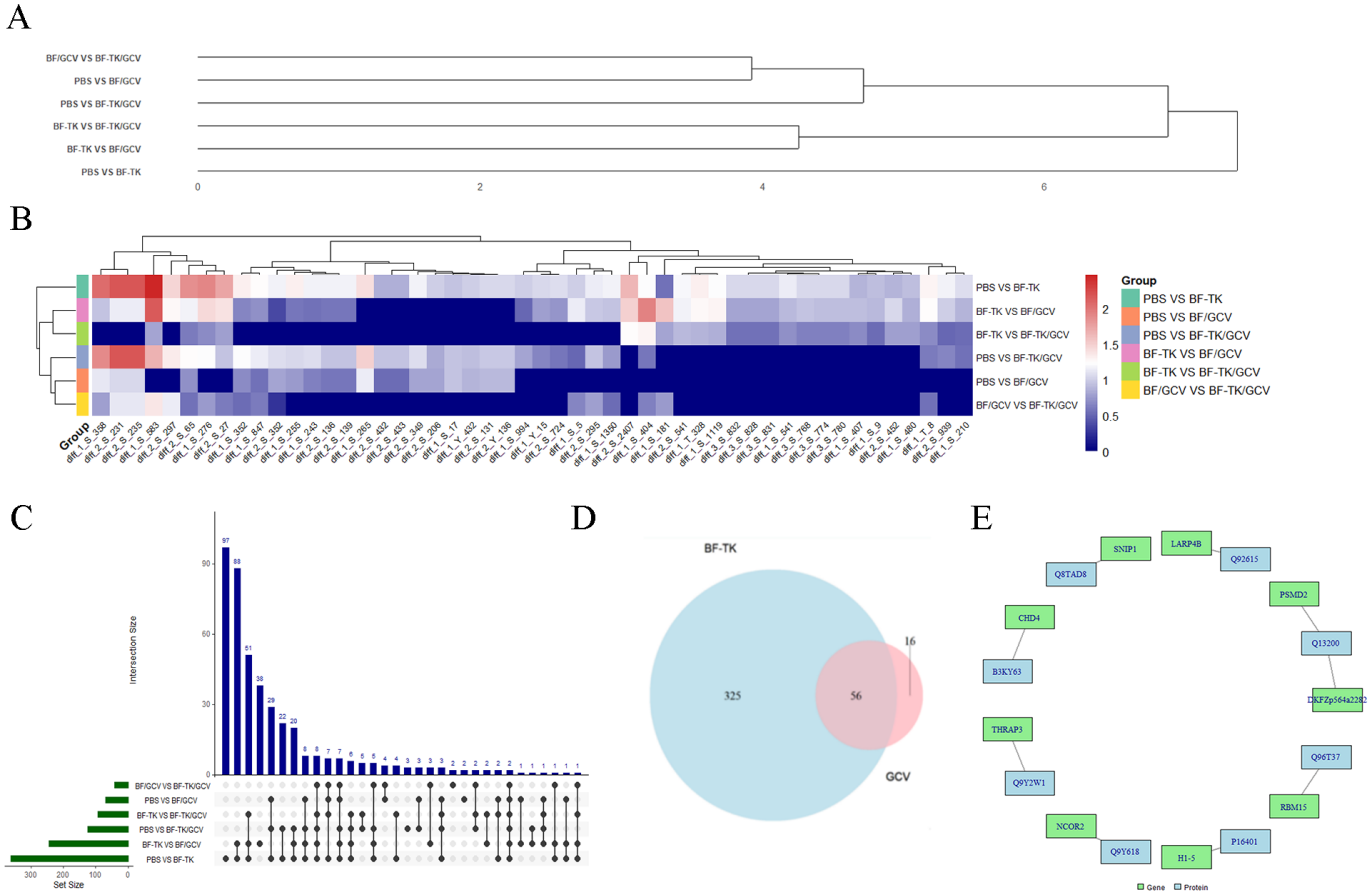
Identification of group-specific differential PIPAs. **(A)** Clustering dendrogram of six pairwise comparisons. **(B)** Heatmap of differentially abundant phosphorylation sites across comparisons (red: increased; blue: decreased). **(C)** UpSet plot showing intersections of significantly regulated phosphorylation sites among experimental groups (bar height: intersection size). **(D)** Venn diagram of phosphorylation sites uniquely regulated by BF-TK. **(E)** Network diagram linking regulatory genes (green) to their corresponding phosphorylated proteins (blue).

Comprehensive phosphoproteomics analysis was performed on the PBS (control group) and the three experimental groups (BF-TK, BF/GCV, and BF-TK/GCV). The sample clustering dendrogram revealed that the BF/GCV vs. BF-TK/GCV comparison group clustered closely with the PBS and BF/GCV comparison groups, while the BF-TK vs. BF-TK/GCV comparison group and the BF-TK vs. BF/GCV comparison group also exhibited similar clustering patterns. In contrast, the PBS vs. BF-TK comparison group formed a separate cluster (**Figure 7A**). The protein heatmap showed that in the PBS vs. BF-TK comparison group, many proteins such as P2RX4 (S315), GATAD2B (S581), and AHNAK (S5110) were marked in red, indicating a high level of phosphorylation. In the BF-TK vs. BF/GCV comparison group, proteins such as CTNND1 (S268) and GATAD2B (S581) also exhibited significantly enhanced phosphorylation. In contrast, the PBS vs. BF-TK/GCV and PBS vs. BF/GCV comparison groups displayed a predominance of blue colors, suggesting lower levels of phosphorylation. Notably, GBF1 (S1318) and EPRS (S886) showed significant phosphorylation enhancement in the PBS vs. BF-TK comparison group (**Figure 7B**).

The clustering analysis results indicated that the PBS vs. BF-TK comparison group and the PBS vs. BF/GCV comparison group exhibited similar clustering patterns, suggesting that BF-TK and BF/GCV treatments may induce similar phosphorylation profiles, with BF acting as a common factor. Notably, the PBS vs. BF-TK/GCV comparison group formed its own distinct cluster, indicating that the activation of GCV in the BF-TK/GCV combined treatment induced a unique phosphorylation regulatory mechanism, differing significantly from the changes observed with BF-TK or BF/GCV treatments alone. The BF-TK vs. BF/GCV comparison group and BF-TK vs. BF-TK/GCV comparison group clustered together, suggesting that the addition of GCV might trigger similar phosphorylation regulatory patterns. However, the independent clustering of the BF/GCV vs. BF-TK/GCV comparison group reflects that the presence of BF-TK has a significant impact on GCV-induced phosphorylation modifications.

The clustering position of the BF-TK vs. BF/GCV comparison group suggests that these two treatments may affect a series of distinct phosphorylation sites or signaling pathways. Notably, the BF-TK/GCV combined treatment likely activated some unique cellular response mechanisms, and the phosphorylation proteins influenced by BF-TK/GCV alone included NCOR2 (S1858), SNIP1 (S52, S54), LARP4B (S718), KIAA1440 (S659), HIST1H1B (S2), CLASP1 (S1509), RAB3IP (S672), and PSMC1 (S16). These findings offer guidance for optimizing tumor treatment strategies.

Both the PBS vs. BF-TK and PBS vs. BF/GCV comparison groups exhibited approximately 300 significantly differential phosphorylation sites, which correlates with their close clustering relationships in the analysis. This suggests that BF-TK and BF/GCV treatments induce broad and similar phosphorylation modification patterns compared to the PBS control group. Notably, the PBS vs. BF-TK/GCV comparison group displayed slightly fewer significantly differential phosphorylation sites. This phenomenon reflects the unique regulatory effects induced when BF-TK and GCV are used together, possibly due to interactions or antagonistic effects between the two treatments (**Figure 7C**).

The analysis also revealed some unique phosphorylation sites, such as NCOR2(S1858), SNIP1(S52, S54), LARP4B(S718), KIAA1440(S659), HIST1H1B(S2), CLASP1(S1509), RAB3IP(S672), and PSMC1(S16), which were only observed in specific comparison groups. These sites may represent treatment-specific regulatory mechanisms and provide valuable insights into the unique biological effects of different treatment combinations.

At the same time, the unique protein phosphorylation in each group was demonstrated. A Venn diagram (**Figure 7D**) was used to investigate the phosphorylation sites regulated uniquely by each group. The analysis revealed that the BF-TK group regulated 269 phosphorylation sites, the GCV group regulated 16 phosphorylation sites, and 56 phosphorylation sites were regulated by all groups in common.

Furthermore, a PPI network analysis was performed for all phosphorylated protein sites expressed across the four groups, including the experimental groups (BF-TK, BF/GCV, BF-TK/GCV) and the control group (PBS). The analysis was conducted using the STRING database, and the results were visualized with Cytoscape (**Figure 7E**). The common phosphorylation sites between groups represent sites with inconsistent phosphorylation scores across the groups. The PPI enrichment displayed in the constructed PPI networks indicates rich interactions, suggesting that these proteins are at least biologically related as a group.

### Pathway and core protein identification

**Figure 8:**
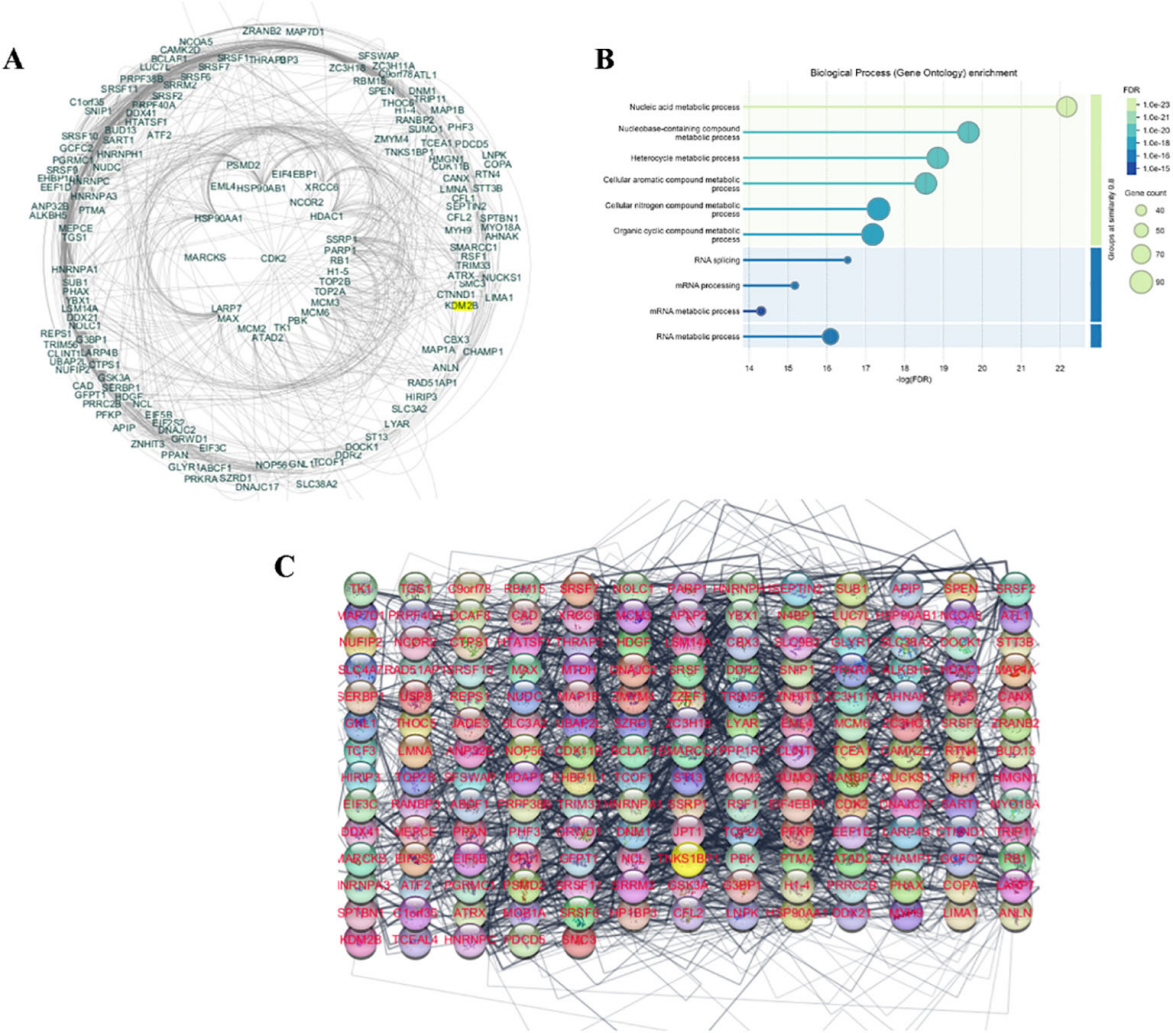
Construction of differential phosphoprotein networks. **(A)** PPI network of all differentially abundant phosphoproteins (nodes: proteins; edges: STRING-predicted interactions). **(B)** Top 10 GO terms enriched in the PPI network shown in **(A, C)** PPI network of BF-TK/GCV-regulated phosphoproteins (red: increased abundance; blue: decreased abundance).

Functional and pathway analysis of the significantly altered phosphorylation sites revealed that in the PPI network of total PIPAs across the four groups (**Figure 8A**), 236 GO terms were significantly enriched (FDR < 0.05), and 95 were highly significant (FDR < 0.01). The top ten GO terms were visualized (**Figure 8B**). DBSCAN clustering analysis was performed to explore the major biological processes involved, resulting in multiple protein clusters and their functional descriptions.

The most prominent cluster was an mRNA splicing-related cluster, which contained 11 proteins involved in mRNA processing and UDP-mediated mRNA stability complexes, including HNRNPC, SRSF10, HNRNPA3, SRSF2, SRSF11, SRSF7, HNRNPA1, HNRNPH1, SRSF1, SRSF6, and SRSF9. The second cluster was a cell cycle-related cluster containing 10 proteins involved in the regulation of DNA replication initiation and activation of the pre-replication complex, including MCM2, MCM3, NCOR2, HDAC1, CDK2, RB1, MCM6, SPEN, CDK3, and HI-4. The third cluster was associated with the U2-type pre-catalytic spliceosome and contained 4 proteins: SNIP1, SFMM2, SART1, and BUD13. These protein clusters cover a broad range of essential cellular processes from mRNA processing, cell cycle regulation, to translation initiation, highlighting the extensive participation and regulatory role of these protein clusters in various important biological functions within the nucleus, as identified through the DBSCAN clustering method.

The PIPAs regulated by both BF-TK and BF-TK/GCV include CANX, MMTAG2, HNRNPA3, LMNA, EIF4EBP1, LYAR, PPP1R7, SRRM2, CTNND1, SMAD2, DNM1, PGRMC1, SRRM2, CLINT1, HNRNPH1, RBM15, PRPF40A, and THRAP3. Among these, CANX, MMTAG2, HNRNPA3, LMNA, EIF4EBP1, PPP1R7, SRRM2, CTNND1, SMAD2, DNM1, PGRMC1, SRRM2, and THRAP3 are dephosphorylated, while LYAR, CLINT1, HNRNPH1, RBM15, and PRPF40A are phosphorylated. Except for SMAD2, which is phosphorylated at threonine (pT), all other protein modifications are phosphorylation at serine (Ps) (**Figure 8C**).

### Exploration of transcriptomic level in TK/GCV system

**Figure 9:**
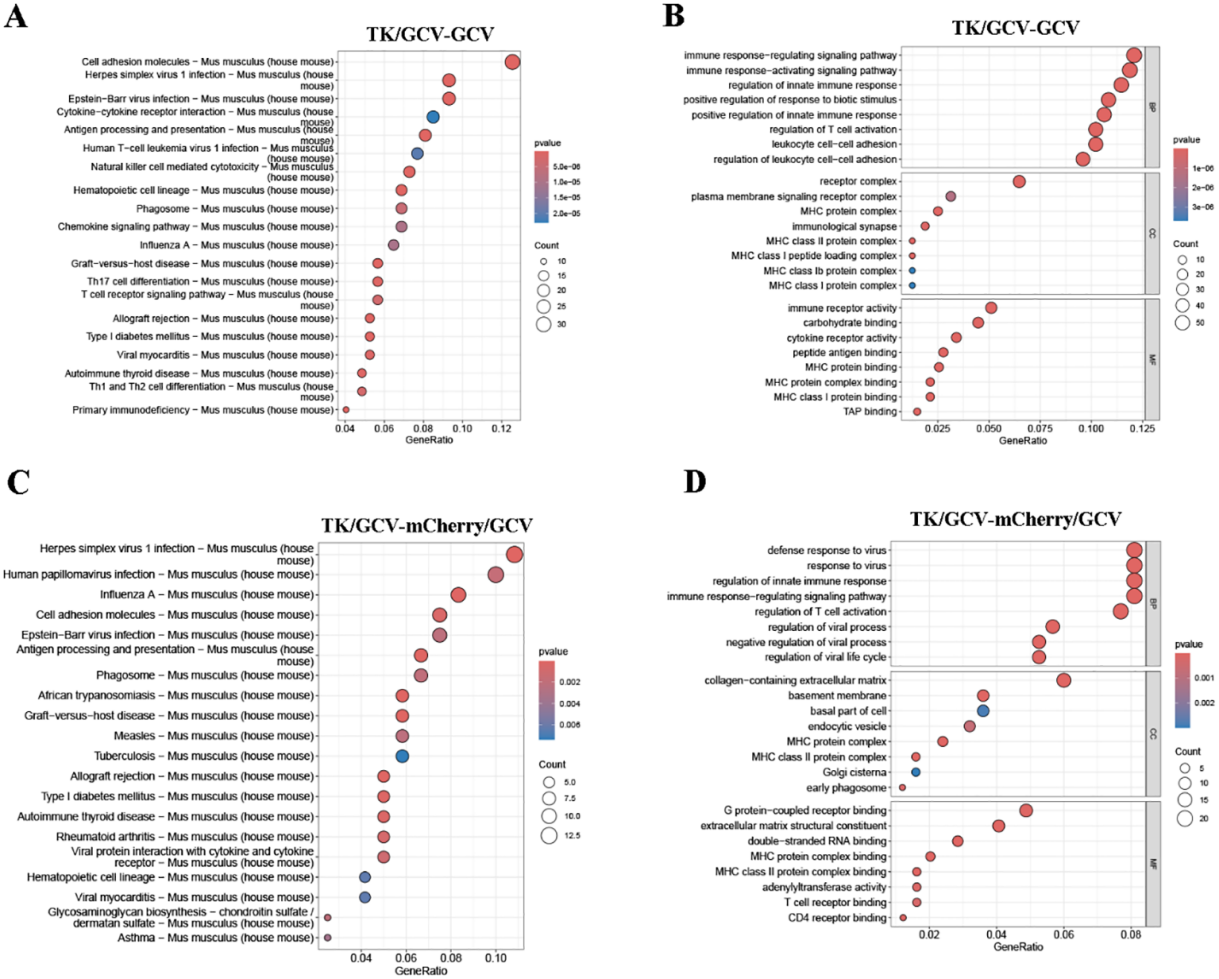
Enriched signaling pathways in the transcriptome of TK/GCV. **(A, B)** KEGG **(A)** and GO **(B)** enrichment analyses of BF-TK/GCV vs. GCV. **(C, D)** KEGG **(C)** and GO **(D)** enrichment analyses of BF-TK/GCV vs. pcDNA3.1-mCherry/GCV.

Next, we explored the potential regulatory mechanisms of the TK/GCV system at the transcriptomic level. We found that the KEGG pathways enriched in the TK/GCV group compared to the GCV group included Herpes simplex virus 1 infection, Cytokine-cytokine receptor interaction, Antigen processing and presentation, Human T-cell leukemia virus 1 infection, Natural killer cell-mediated cytotoxicity, Th17 cell differentiation, T cell receptor signaling pathway, Th1 and Th2 cell differentiation, and Primary immunodeficiency (**Figure 9A**). GO analysis indicated that BP enrichment involved immune response-regulating signaling pathway, immune response-activating signaling pathway, regulation of innate immune response, positive regulation of innate immune response, and regulation of T cell activation, while CC enrichment included MHC protein complex, immunological synapse, MHC class II protein complex, and MHC class I peptide loading complex. MF enrichment included immune receptor activity, MHC protein binding, and MHC protein complex binding (**Figure 9B**). All of this suggests that our TK/GCV system is closely related to the activation of immune signaling pathways.

In addition, compared to the mcherry/GCV group, the KEGG pathways enriched in the TK/GCV group included Transcriptional misregulation in cancer, HIF-1 signaling pathway, and Viral protein interaction with cytokine and cytokine receptors (**Figure 9C**). The GO analysis of BP and CC enrichment was related to the regulation of various epithelial cell processes, while MF enrichment was associated with immune receptor activity and some kinase signaling pathways (**Figure 9D**). This suggests that as a negative control, the bifidobacterium vector in the bloodstream can activate various cascading signaling pathways. Therefore, the TK/GCV suicide system may influence the tumor immune microenvironment at the transcriptional level. Whether these transcriptional changes translate into functional immune remodeling remains to be determined in future studies.

### Immunohistochemical validation of related protein expression

**Figure 10:**
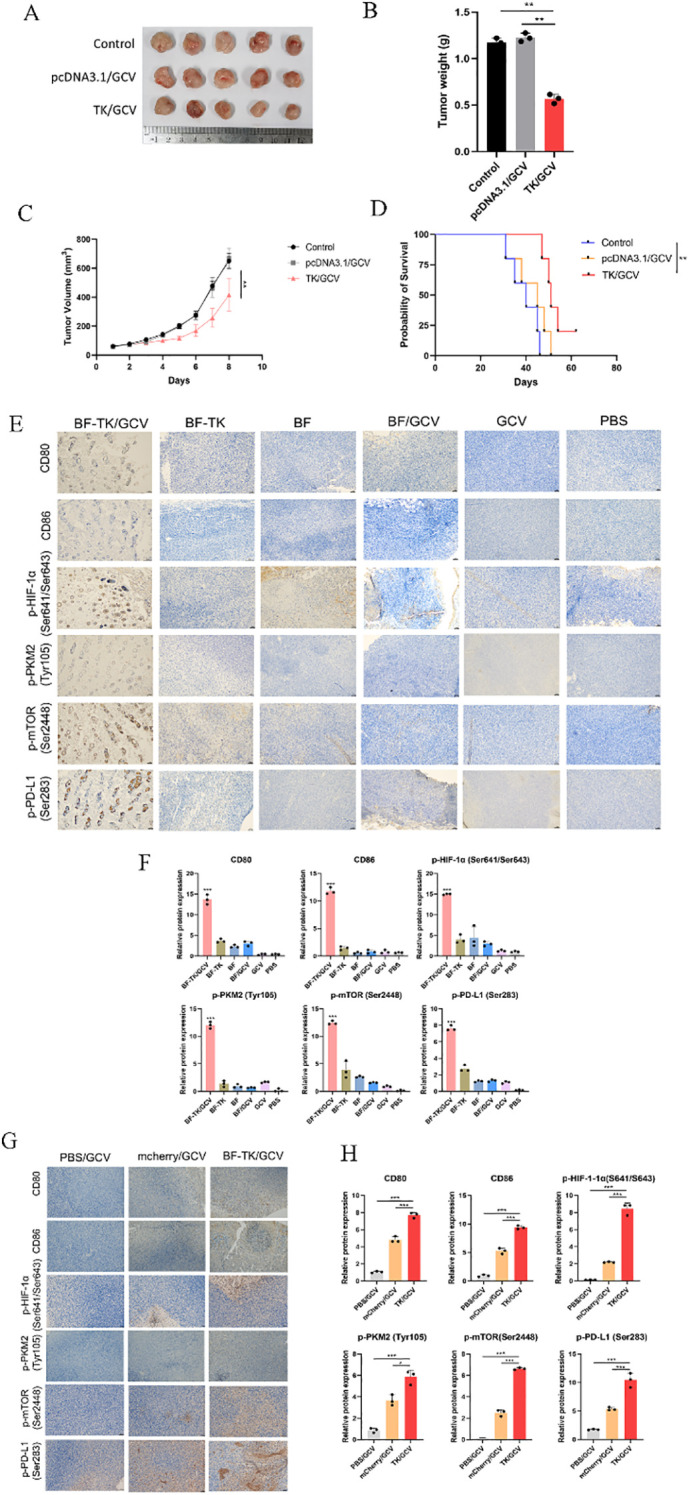
Immunohistochemical validation in multiple mouse solid tumor models. **(A–C)** Tumor volume, weight, and growth curves (eight doses). **(D)** Kaplan-Meier survival curves. **(E, F)** IHC quantification of phospho-HIF-1α, phospho-PKM2, phospho-mTOR, and phospho-PD-L1 in immune-deficient (nude) mouse xenografts (n=6). **(G, H)** IHC quantification of the same phosphoproteins plus CD80 and CD86 in immune-competent (C57BL/6J) MC38 syngeneic tumors (n=5). *Data are shown as mean ± SEM. *p < 0.05, **p < 0.01, ***p < 0.001 (one-way ANOVA)*.

The *in vivo* efficacy of BF-TK/GCV therapy was evaluated in immunocompetent mice bearing MC38 xenografts, addressing a gap left by our previous studies that were limited to immunodeficient models. Treatment significantly inhibited tumor growth, as evidenced by reduced tumor volume on day 8 post-injection (**Figures 10A–C**). Furthermore, survival analysis demonstrated that BF-TK/GCV treatment significantly prolonged the overall survival of tumor-bearing mice, as shown by Kaplan–Meier curves (**Figure 10D**).

To visually assess the phosphorylation status of selected proteins *in vivo*, we performed immunohistochemistry (IHC) on tumor tissues. We selected immune-deficient (nude) mouse xenograft tumors and mouse-derived immune-competent xenograft tumors for this analysis. As shown in **Figures 10E, F**, in the human-derived xenograft tumor, the BF-TK/GCV group exhibited the highest immunoreactivity for phospho-HIF-1α (S641/643), phospho-PKM2 (Y105), phospho-mTOR (S2448), and phospho-PD-L1 (S283). These IHC findings are supportive of the phosphoproteomic data showing increased abundance of these specific phosphosites. However, IHC is semi-quantitative and antibody-dependent; therefore, these results should be considered complementary rather than definitive validation. In the immune-competent C57 mouse model (**Figures 10G, H**), we also observed increased immunoreactivity for several phosphorylated proteins and increased expression of CD80 and CD86.

These results suggest that TK/GCV can alter the tumor immune microenvironment in mouse solid tumors and activate phosphorylation of multiple signaling pathways.

## Discussion

Previous studies have shown that BF-TK/GCV exhibits strong anti-tumor properties in solid tumors through multiple mechanisms (11, 19–21). The phosphoproteomic profiling of tumor cells treated with BF-TK/GCV provides new insights into the mechanisms of action of BF-TK/GCV. To explore the relationship between BF-TK/GCV and tumor growth regulation, we employed an efficient method combining TiO2 phosphopeptide enrichment and HPLC-MS/MS analysis for phosphoproteomic analysis. At the same time, to validate the potential mechanisms of BF-TK/GCV gene therapy, we performed transcriptomic sequencing. To confirm the feasibility of this system as a gene therapy approach, we conducted immunohistochemical validation.

In this study, based on different groups of PBS, BF-TK, BF/GCV, and BF-TK/GCV, we identified 5814 phosphopeptides, involving 5592 phosphosite and 2386 PIPAs, among which over 40% of the PIPAs contained phosphoserine sites. This is consistent with previous studies (35, 36), highlighting the dominant role of serine phosphorylation in cellular signal transduction. More importantly, compared to the BF-TK and BF/GCV groups, we observed a significant number of DAPs after BF-TK/GCV treatment. This not only indicates that phosphorylation plays a key role in mediating the anti-tumor effects of BF-TK/GCV but also reflects the dynamic reorganization of the cellular phosphorylation network. The extensive changes in phosphorylation patterns reveal the central role of phosphorylation regulation in tumor cell responses to treatment, providing important insights into the mechanisms of the BF-TK/GCV system and offering a basis for developing more precise targeted therapeutic strategies.

PIPAs co-regulated by BF-TK and BF-TK/GCV include CANX, MMTAG2, HNRNPA3, LMNA, EIF4EBP1, LYAR, PPP1R7, SRRM2, CTNND1, SMAD2, DNM1, PGRMC1, SRRM2, CLINT1, HNRNPH1, RBM15, PRPF40A, THRAP3, among others. These proteins may regulate certain mechanisms through phosphorylation modifications to exert anti-tumor effects, but further investigation is needed to explore their functions and mechanisms of action.

The phosphorylation of proteins specific to the BF-TK/GCV response may reflect important functions in the action of BF-TK/GCV. Several cancer-related signaling pathways were enriched, including p53, cell cycle, and DNA replication. For example, MAP2K2 was significantly dephosphorylated in the enriched pathway analysis, and MAP2K2 is associated with colorectal cancer (36). C8orf48 inhibits the MAPK signaling pathway in colorectal cancer, and the expression of C8orf48 is negatively correlated with MAP2K2 in normal colorectal tissue and with MAP2K2 in colorectal cancer tissue. Overexpression of C8orf48 reduces the proliferation, migration, and invasion of colorectal cancer cells (37). The dephosphorylation of MAP2K2, leading to its inactivation, may increase the expression of C8orf48, which could further enhance the inhibition of proliferation, migration, and invasion in colorectal cancer cells. This suggests that BF-TK/GCV may regulate these signaling pathways to exert its anti-tumor effects, consistent with our previous findings. Additionally, we are interested in the HIF-1 signaling pathway. HIF-1α is a transcription factor under hypoxic conditions, widely present in mammals and humans (38), and it can form different signaling pathways with various proteins to regulate cellular processes such as cell growth (39), proliferation (40), metastasis (41), and apoptosis (42). Numerous studies have reported the relationship between the HIF-1 signaling pathway and tumor metastasis. It has been reported that ERBIN promotes colorectal cancer metastasis through miR-125a-5p and miR-138-5p/4EBP-1-mediated translation of HIF-1α (43), while PTBP3 promotes tumor cell proliferation, migration, invasion *in vitro*, and tumor growth and metastasis *in vivo*. HIF-1α is responsible for PTBP3-induced cell migration and invasion, and activation of HIF-1α helps promote tumor cell proliferation, migration, and invasion (44). Therefore, the regulatory mechanism of BF-TK/GCV urgently requires further experimental validation.

After a deep investigation of the phosphoprotein data, we identified phosphoproteins that are specifically regulated by BF-TK/GCV. The NCOR2 protein regulates gene expression by recruiting and activating histone deacetylase 3 (HDAC3) (45). The macrophage inflammatory pathway is strictly regulated by various transcription factors, such as NF-κB, Adenovirus Type 1, PPAR family, LXR, as well as co-activators and co-repressors (NCoR1/NCoR2) (46). Moreover, nuclear co-repressors NCOR1 and NCOR2 interact with various transcription factors, indicating that NCOR1/2 may play a ubiquitous but stage-specific role during development (47). Changes in interactions with HDAC3 or other transcription factors can affect their localization or stability in the nucleus, altering their regulatory ability on specific genes. The phosphorylation of serine residues in NCOR2 affects this regulatory mechanism, leading to its abnormal function, which inhibits normal transcription in tumor cells and suppresses their metastasis. At the same time, it may inhibit genes that promote tumor metastasis. SNIP1 is a nuclear inhibitor of CBP/p300, and its expression levels in specific cell types have important physiological consequences by setting the transcription activation threshold involving CBP/p300 induced by TGF-β (48). EMT regulators such as Snail, Slug, TWIST, SMAD, and SNIP1 have been associated with tumor progression and prognosis in various types of human cancers, including ovarian cancer, breast cancer, colorectal tumors, uterine cancer, hepatocellular carcinoma, esophageal squamous cell carcinoma, gliomas, and oral squamous cell carcinoma (48). Dephosphorylation of SNIP1 enhances its ability to inhibit CBP/p300, thereby suppressing the expression of genes that promote tumor metastasis. This may help inhibit tumor metastasis. LARP4B deletion, in combination with p53 and Nf1 defects, promotes tumor cell growth. LARP4B has been identified as a potential cancer-related gene in other transposon mutagenesis screenings, including liver cancer, colorectal cancer, pancreatic cancer, malignant peripheral nerve sheath tumors, and medulloblastoma. This suggests that LARP4B may be involved in the development of various cancers. The patterns of transposon insertion in gastrointestinal tumors and liver cancer support its role as a tumor suppressor (49). Phosphorylation of LARP4B may activate its activity, enhancing its tumor suppressive functions and potentially inhibiting tumor growth and metastasis.

In the deep analysis of phosphoprotein data, we investigated the differential phosphorylation sites between the comparison groups, revealing the distinct effects of single and combination treatments. This provides new insights into the dynamic regulation of cellular signaling networks. The BF-TK/GCV combination treatment activated signaling pathways that were not triggered by single treatments, suggesting a synergistic effect. This synergy arises from one treatment providing a “kick-start” or “amplification” effect to the other, involving conformational changes or subcellular localization alterations of key proteins. The observed additive and antagonistic effects reflect the complexity and adaptability of cellular signaling networks, which may involve negative feedback regulation or activation of parallel signaling pathways. Phosphorylation events triggered only under strong stimuli indicate a “threshold effect” in the cellular signaling network, which may be a strategy for the cell to filter out environmental noise. Phosphorylation sites with consistently altered trends may represent core response mechanisms of the cell to external stimuli, involving basic survival functions. These findings construct a complex network map of cellular responses, illustrating how BF-TK and GCV influence cellular signal transduction and functions through multiple layers of mechanisms.

In previous studies, our group verified eight proteins related to metastasis through IHC, revealing that the HIF-1 signaling pathway may be an important pathway in the BF-TK/GCV anti-tumor metastasis mechanism (21). Factors such as NF-κB, STAT3, CXCL12/CXCR4, and MSK1 play key roles in the metastasis of various cancers (38, 50–53). The results showed that proteins such as HIF-1A, NF-κB1, VCAM1, CEBPB, CXCL12, and p-CREB1 were phosphorylated in the BF-TK/GCV group, suggesting that BF-TK/GCV may inhibit tumor growth through these proteins. We further validated changes in the expression of CD80, CD86, as well as phosphorylation modifications of PKM2, mTOR, HIF1A, and PD-L1 in multiple *in vivo* models, proving the potential immune functions of BF-TK/GCV gene therapy. This study reveals that BF-TK/GCV may inhibit tumor growth through a multi-layered complex network, including: 1) Phosphorylation regulation of key gene mRNA stability; 2) Cooperative phosphorylation of mRNA processing, cell cycle, and DNA replication-related proteins; 3) Phosphorylation of spliceosome-related proteins altering tumor gene splicing patterns; 4) Activation of the tumor microenvironment and the potential for PD-L1 immune therapy. This multi-layered regulatory mechanism provides important clues for the development of new anti-tumor strategies.

However, several limitations must be acknowledged. First, phosphopeptide abundance changes were not normalized to total protein levels; therefore, observed differences may reflect altered protein abundance rather than true phosphorylation occupancy. Second, direct evidence of functional immune cell infiltration or activation (e.g., by flow cytometry) was not obtained. Third, the MC38 model lacked control groups (e.g., GCV only, BF-TK only) to fully dissect individual treatment contributions. Fourth, future studies should include bacterial burden quantification to establish a clearer link between colonization and therapeutic efficacy. Future studies using genetic or pharmacological perturbation of HIF-1α, mTOR, and PD-L1, are required to determine whether they are necessary and sufficient for the observed antitumor effects.

**Figure 11:**
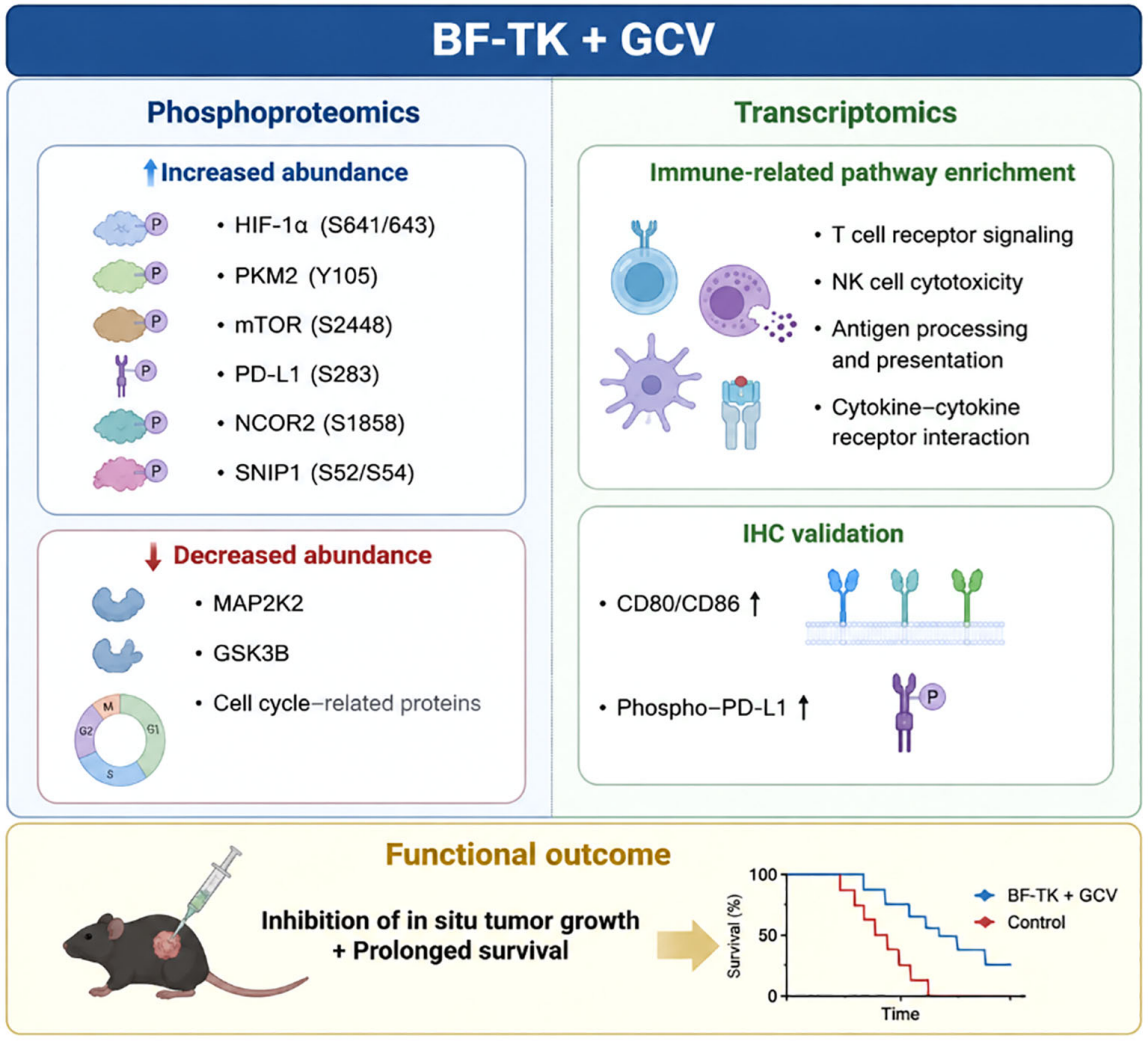
Schematic working model summarizing the key findings of this study.

Despite these caveats, our findings provide a multi-layered resource (phosphoproteomic + transcriptomic + IHC) for understanding how BF-TK/GCV may exert antitumor effects. We identify specific phosphoproteins (e.g., NCOR2, SNIP1, LARP4B) and pathways that warrant mechanistic follow-up. Future studies incorporating total proteome normalization, functional immune profiling, and complete control groups are required to test the hypotheses generated here and to establish causality (**Figure 11**).

## Conclusion

In this study, we demonstrate that BF-TK/GCV treatment modulates the phosphoproteome and transcriptome of solid tumors in a site- and pathway-specific manner. Our phosphoproteomic analysis identified widespread changes in phosphopeptide abundance—including both increased and decreased abundance—particularly in pathways related to the cell cycle, HIF-1 signaling, and AMPK signaling. Concurrent transcriptomic sequencing revealed enrichment of immune-related pathways, suggesting a transcriptional association with potential immune microenvironment modulation.

## Data Availability

The RNA-seq and Proteomics data have been deposited in BIG Sub under the accession code OMIX017292 (https://ngdc.cncb.ac.cn/omix/release/OMIX017292).
