# Supplementary material for: Bifidobacterium infantis-mediated HSV-TK/GCV therapy modulates the tumor microenvironment through site-specific phosphorylation of HIF-1α, mTOR, and PD-L1
Source: Front Oncol. 2026 Jun 22;16:1849164. doi: 10.3389/fonc.2026.1849164 (PMC13334318; doi:10.3389/fonc.2026.1849164)
Supplement: Supplementary file 1 [file DataSheet1.docx]

**Bifidobacterium infantis‑mediated HSV‑TK/GCV therapy modulates the tumor microenvironment through site‑specific phosphorylation of HIF‑1α, mTOR, and PD‑L1**


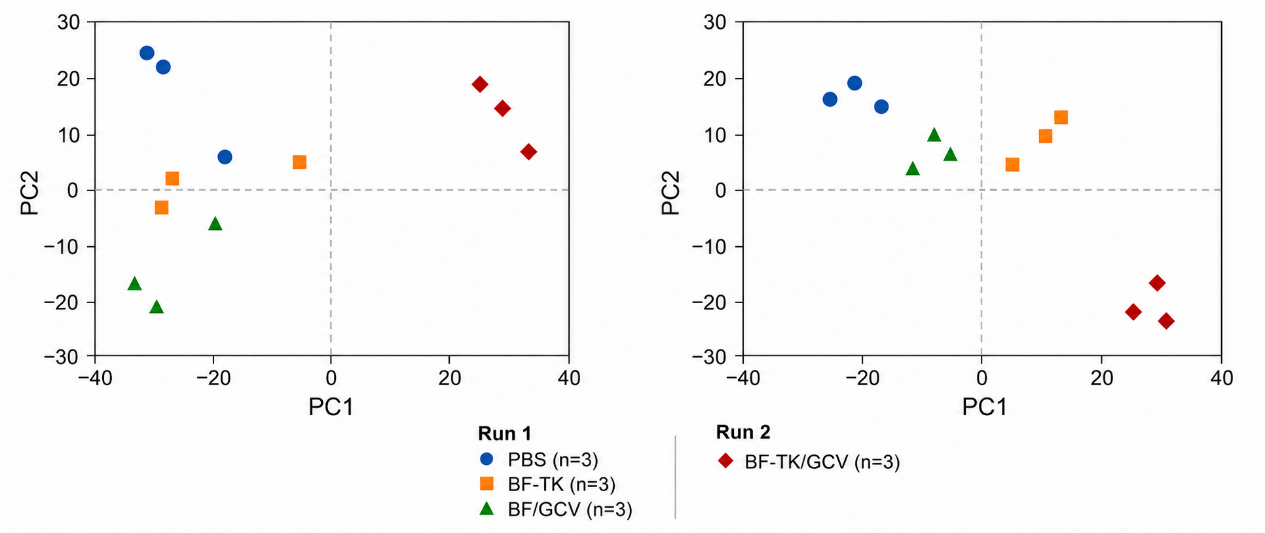


**Figure S1. Principal component analysis (PCA) of phosphoproteomics samples before and after batch correction.**

(A) PCA before batch correction. Samples separate primarily by TMT run (Run 1: PBS (n=3), BF-TK (n=3), BF/GCV (n=3); Run 2: BF-TK/GCV (n=3)), indicating a batch effect.

(B) PCA after ComBat batch correction. Samples now cluster primarily by treatment group (PBS (n=3), BF-TK (n=3), BF/GCV (n=3), BF-TK/GCV (n=3)) rather than by TMT run, indicating successful mitigation of batch effects.
